# Chemical and Rheological Evaluation of the Ageing Behaviour of High-Content Crumb Rubber Asphalt Binder

**DOI:** 10.3390/polym16213088

**Published:** 2024-10-31

**Authors:** Zhilian Ji, Zhibin Wang, Lei Feng, Peikai He, Song Li

**Affiliations:** 1 School of Traffic and Transportation, Shijiazhuang Tiedao University, Shijiazhuang 050043, China; jizhilian920@163.com; 2 Taihang Urban and Rural Construction Group Co., Ltd., Shijiazhuang 050222, China; 3 Hebei Key Laboratory of Traffic Safety and Control, Shijiazhuang 050043, China

**Keywords:** ageing asphalt, high-content crumb rubber, chemical property, rheological property, correlation

## Abstract

High-Content Crumb Rubber Asphalt (HCRA) binder improves road performance and address waste tyre pollution, yet its ageing behaviour is not fully understood. In this study, 70# neat asphalt binder and HCRA with rubber contents of 35% and 50% were selected and aged through the Thin Film Oven Test (TFOT) and Pressure Ageing Vessel (PAV) tests. FTIR (Fourier Transform Infrared Spectroscopy) and DSR (Dynamic Shear Rheometer) were employed to investigate their chemical composition and rheological properties. The FTIR results show that HCRA’s chemical test results are similar to those of 70#, but HCRA is more susceptible to ageing. I(C=C) strength decreases with age. The DSR results show that HCRA outperforms 70# neat asphalt binder in terms of viscoelasticity, high temperature performance and fatigue resistance, and exhibits greater resistance to ageing. The ageing index (AI) was obtained through a calculation using the formula, and overall, 70# neat asphalt binder is more sensitive to ageing behaviour and less resistant to ageing, and HCRA is particularly outstanding for fatigue resistance. A strong correlation is observed between chemical composition and some rheological property indicators. Therefore, we are able to predict the rheological properties using chemical composition indicators. This study provides insight into the ageing behaviour of a neat asphalt binder and an HCRA binder and demonstrates that the HCRA binder outperforms conventional asphalt in several performance areas. It also provides theoretical support for the consumption of waste tyres to prepare high content crumb rubber asphalt.

## 1. Introduction

Recently, rapid developments in the automobile industry have promoted convenient travel conditions. However, this growth has also resulted in the production of a significant number of waste tyres and other types of industrial waste [1], which have caused increasingly severe environmental pollution [2,3]. Waste tyres have been used as crumb rubber modifiers in asphalt binder to make crumb rubber asphalt (CRA) binder, which is considered to be a solution to the problem of environmental pollution. This utilization approach contributes to the consumption of large quantities of waste tyres and enhances the performance of asphalt binder [4]. Therefore, crumb rubber has become common in the road paving industry because it can significantly improve the physical properties of asphalt binder and provide environmental benefits [5]. The conventional CRA preparation method involves adding crumb rubber to asphalt binder directly, followed by high-speed shearing. As a result, the crumb rubber undergoes physical swelling as well as partial chemical dissolution, without forming a homogeneous system with the asphalt binder. This process affects the performance of the CRA and limits the amount of crumb rubber that can be used, which is typically approximately 20% relative to the mass of the asphalt binder [6]. Therefore, it is necessary to add modifiers to prepare a high-content (>20%) crumb rubber asphalt (HCRA) binder. The main HCRA production mechanism is desulphurization, which reduces the molecular weight of the crumb rubber [7]. In addition, the swelling and degradation of the crumb rubber positively affect the rheological and anti-ageing properties of CRA, where the swelling process of the crumb rubber is a multi-physical phenomenon including mass diffusion and volume expansion (mechanical deformation) [8]. During the swelling process, the crumb rubber adsorbs the lighter components of the asphalt binder (aromatics, etc.), which increases the crumb rubber volume by 3–5 times [9]. Degradation can be considered to involve a combination of devulcanization and depolymerization of the crumb rubber in the HCRA [10]. It has been shown that increasing the degree of degradation and regeneration of crumb rubber can significantly increase its content in asphalt binder and reach the maximum possible rubber content, making the dispersion of crumb rubber in asphalt binder more uniform, and improving the flowability during processing and stability during high-temperature storage [11].

Asphalt binders often undergo ageing and degradation reactions during use, especially under the strong influence of heat, oxygen, sunlight and time; these reactions are irreversible [12,13]. The ageing of asphalt binder is currently well researched. The ageing process is characterized by a series of polymerization, oxidation and volatilization reactions [14] and even internal structural changes [15]. The ageing of asphalt binder can be divided into two stages: short-term ageing and long-term ageing [16]. Short-term ageing occurs during mixing, transport and paving, mainly due to the loss of volatile components and rapid oxidation at high temperatures. Long-term ageing occurs over the lifetime of an asphalt pavement and is related to continued oxidation, hardening and UV radiation [17]. Aged asphalt binder becomes hard and fragile, and its water stability, low-temperature cracking properties and fatigue resistance decrease [18]. Accelerated ageing of asphalt binder directly leads to the deterioration of pavement performance and affects the durability of the pavement [19]. Chemical studies of asphalt binder ageing commonly use Fourier transform infrared spectroscopy (FTIR) to quantify the extent of ageing in the asphalt binder at the molecular level. The results show that indices based on the carbonyl (C=O) and butadiene (C=C) contents are suitable for evaluating the effects of ageing on 70# neat asphalt binder and modified asphalt binder, respectively [20]. Dynamic shear rheometer (DSR) analyses of the rheological properties of asphalt binder have revealed that ageing can cause the curve in the Black diagram to shift to a lower phase angle, it is a specific form of graphical representation of the master curve, where the horizontal and vertical coordinates are in (log(G*)) form. With this graph, it is possible to reflect the viscoelastic properties of asphalt. As the shape of this plot changes to straight lines and exhibits reduced curvature, these changes indicate that the material response tends to be more brittle [21]. At the same time, according to the complex shear modulus, phase angle and rutting factor, SBS (styrene–butadiene–styrene) can effectively improve the high-temperature deformation resistance of ACR (activated crumb rubber powder)/SBS asphalt [22]. The incorporation of polymer modifiers has been shown to significantly increase the energy storage modulus and loss modulus of the composites and reduce the magnitude of the energy storage modulus with temperature by several orders of magnitude [23].

Due to the complexity of the internal structure of CRA, its ageing mechanism is also complex. During ageing, crumb rubber and asphalt binder undergo a variety of reactions such as polymerization, condensation, cleavage and cross-linking. Due to the complex internal composition and structure of CRA, these reactions occur at different stages and degrees of ageing [24]. The thermo-oxidative ageing process of CRA is similar to that of 70# neat asphalt binder, but the continuous interaction between crumb rubber and asphalt binder increases the complexity of studying this process. The effect of crumb rubber in CRA is mainly based on interaction mechanisms: crumb rubber absorbs the light components of the asphalt binder and simultaneously releases the oily components, carbon black and inorganic fillers into the asphalt binder [25,26]. The effect is influenced by the type, particle size and composition of the crumb rubber; the source and properties of the asphalt binder; the compatibility between the crumb rubber and asphalt binder; and the mixing temperature, mixing time and shear rate during preparation [27]. The solubilized crumb rubber protects lightweight components, whereas the released carbon black improves the ageing resistance of the asphalt binder [28]. The crumb rubber releases antioxidants into the asphalt binder via desulphurization and depolymerization; degraded crumb rubber acts as a softener, while increased ageing promotes the desulphurization and degradation of the crumb rubber [29]. The reticulated polymeric chain structure of crumb rubber is also effective in blocking oxygen molecules [30]. Therefore, it is generally believed that the addition of crumb rubber can effectively improve the anti-ageing properties of CRA and that a greater degree of interaction between the crumb rubber and asphalt binder corresponds to a greater resistance of CRA to thermal and oxygen ageing. However, it has been revealed that ageing causes hardening of the asphalt binder and desulphurization of the crumb rubber, which weakens the efficacy of the crumb rubber, and coupling reactions between the asphalt binder and crumb rubber cause various pavement diseases [31,32,33]. Therefore, the specific ageing behaviour of CRA, which affects the road performance of asphalt binder, remains a focal issue.

The content of crumb rubber significantly affects the performance of the asphalt binder. Increasing the dosage of rubber powder makes the asphalt surface rougher, increases the adhesion area and enhances the crack resistance of the asphalt mixture [34]. Ghavibazoo et al. [35] reported that the addition of a higher content of crumb rubber (20% crumb rubber relative to the mass of the asphalt binder) improved the low-temperature performance of asphalt binder after ageing compared to that with the addition of 0 and 10% crumb rubber. However, It has been showed that HCRA (with approximately 50% crumb rubber by mass of asphalt binder) had advantages compared to CRA with a 20% crumb rubber content [7]. Wang et al.’s [36] study found that the anti-ageing properties of HCDRA (high-content degraded crumb rubber–modified asphalt) were improved due to the addition of neat asphalt binder and 20DRA (degraded crumb rubber-modified asphalt). On the one hand, the use of HCRA can improve the recycling rate of crumb rubber, reduce the environmental problems caused by waste tyres and save costs. On the other hand, the low-temperature performance of asphalt binders is improved by the use of HCRA [7,37,38]. Huang et al. [39] created HCRAs with three different crumb rubber contents using the wet method. They constructed a master curve based on the time–temperature superposition method for the ageing time shift and found that the addition of crumb rubber effectively increased the elasticity of the asphalt binder, which improved its rutting resistance. Guo et al. [40] proposed a preparation method for a high-volume crumb rubber-modified asphalt binder and analyzed its high-temperature stability, low-temperature cracking resistance, ageing resistance and other properties. The results showed that HCRA had a better low-temperature cracking resistance and ageing resistance and a slightly lower high-temperature stability than CRA. Huang et al. [41] also investigated the effect of crumb rubber content on the rheological properties of HCRA after long-term ageing. They found that, for a highly incompatible asphalt binder, the crumb rubber content significantly affected its rheological properties, with a generally proportional effect. However, for a compatible asphalt binder, the addition of crumb rubber increased the elasticity of the aged asphalt and reduced the degree to which the viscosity increased. Guo et al. [34] investigated the low-temperature performance of HCRA and found that increasing the crumb rubber content made the surface of the asphalt binder rougher and reduced the deformation capacity of the asphalt binder. Using FTIR, Wang et al. [42] found that, as the ageing progressed, the crumb rubber in the HCRA continued to undergo desulphurization and degradation reactions, and ageing increased the compatibility of HCRA while decreasing its integrity and fatigue resistance. Additionally, Xiao et al. [43] studied HCRA before and after ageing using a DSR and a bending beam rheometer (BBR) combined with microscopic analysis. It has been presented that HCRA exhibited excellent high- and low-temperature performance and fatigue resistance compared to 70# neat asphalt binder, and the best performance was achieved with a crumb rubber content of 40% relative to the mass of the asphalt binder.

Therefore, the mechanisms of the ageing behaviour of HCRA remain complex and not completely clear. Further exploration and investigation are needed. The purpose of this study is to understand the ageing behaviour of HCRA comprehensively. In this study, two HCRAs with crumb rubber contents of 35% and 50% relative to the mass of asphalt binder were selected. The effects of thermal oxidation ageing on the chemical composition and rheological properties of 70# neat asphalt binder and HCRAs were investigated. This study provides insights into the ageing behaviour of HCRA. The difference in the ageing responses of HCRA and 70# neat asphalt binder was also discussed.

## 2. Research Objectives

In order to clarify the ageing behaviour of 70# neat asphalt binder and HCRA binders after different ageing degrees, a FTIR test and DSR test were mainly used. The main research objectives are as follows:

(1) Investigate the changes in the chemical composition of asphalt binders before and after ageing using FTIR testing.
(2) Evaluate the rheological properties of asphalt binders before and after ageing using the DSR test.
(3) Discuss the ageing susceptibility of asphalt binder using ageing indices.
(4) Explain the correlation between chemical composition indicators and rheological properties.

## 3. Materials and Methods

### 3.1. High-Content Crumb Rubber Asphalt (HCRA) Binder

Three types of asphalt binders were selected for this study: 70# neat asphalt binder and two HCRA binders. The two HCRA binders were prepared with 70# neat asphalt binder and 40-mesh crumb rubber particles. The properties of the crumb rubber are shown in Table 1. The crumb rubber content was calculated using Equation (1). The HCRA specimens with crumb rubber contents of 35% and 50% by mass relative to the asphalt binder were denoted HCRA35% and HCRA50%, respectively. In addition, the 70# neat asphalt binder was denoted as 70# in this study. To ensure the homogeneity of the HCRA specimens and the accuracy of the experimental results, the HCRAs were prepared in an asphalt plant. The produced asphalt binders were used to conduct experiments in the laboratory. The crumb rubber was desulphurized before HCRA preparation. The desulfurization process involves mixing and reacting crumb rubber with chemical additives at a specific temperature and pressure. Chemical reagents are used to directionally catalyze the cleavage of cross-linking bonds in vulcanized crumb rubber and stabilize the fracture points, thereby achieving the desulfurized crumb rubber. The basic technical properties of 70#, HCRA35% and HCRA50% are presented in Table 2.

(1) $$\mathrm{crumb}\;\mathrm{rubber}\;\mathrm{content}=\frac{\mathrm{mass}\;\mathrm{of}\;\mathrm{crumb}\;\mathrm{rubber}\;}{\mathrm{mass}\;\mathrm{of}\;\mathrm{the}\;\mathrm{resulting}\;\mathrm{rubber}\;\mathrm{asphalt}\;\mathrm{binder}}$$

### 3.2. Ageing of 70# and HCRAs

**Figure 1:**
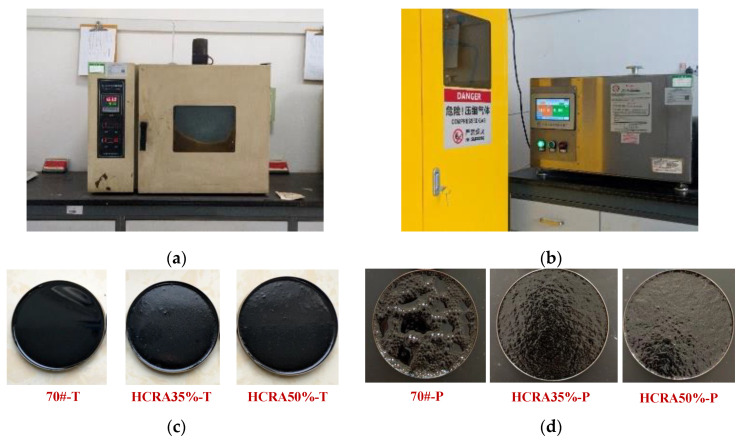
Ageing test equipment and aged asphalt binder specimens. (**a**) Thin-film oven; (**b**) pressure ageing vessel; (**c**) short-term-aged specimens; (**d**) long-term-aged specimens.

In this study, the thin-film oven test (TFOT) and pressure ageing vessel (PAV) test were used to conduct short-term and long-term ageing tests on 70#, HCRA35% and HCRA50%, respectively. During the TFOT, the temperature was maintained at 163 °C ± 1 °C for 5 h. In the PAV test, the air pressure was maintained at 2.1 MPa ± 0.1 MPa and the temperature was controlled at 90~110 °C for 20 h ± 10 min. Asphalt binder specimens with different degrees of ageing were obtained. The 70#, HCRA35% and HCRA50% asphalt binder specimens after TFOT were demoted as 70#-T, HCRA35%-T and HCRA50%-T, which represent the short-term-aged asphalt binder specimens, respectively. In addition, the short-term-aged 70#, HCRA35% and HCRA50% after PAV test were demoted as 70#-P, HCRA35%-P and HCRA50%-P, respectively. They are the asphalt binder specimens after long-term ageing. The equipment used for short-term ageing and long-term ageing is shown in Figure 1a,b. Specimens of the three asphalt binders after short-term ageing and long-term ageing are shown in Figure 1c,d.

## 4. Experimental Methods

In this study, FTIR was used to investigate the changes in the chemical composition of 70#, HCRA 35% and HCRA 50% after different ageing conditions. In addition, the DSR test was conducted to investigate the rheological properties of 70#, HCRA 35% and HCRA 50% before and after ageing. The frequency sweep test, MSCR test and LAS test were used, respectively, to evaluate changes in the viscoelastic properties, high-temperature performance, and fatigue properties of 70#, HCRA35% and HCRA50% at different degrees of ageing.

### 4.1. FTIR

Attenuated total reflectance FTIR spectra of the asphalt binders were obtained in the wavelength range of 4000 cm^−1^ to 500 cm^−1^ with a scanning resolution of 4 cm^−1^. The measured FTIR spectra were quantitatively analyzed to determine the changes in functional groups in the asphalt binder at various ageing stages. Every specimen was subjected to three tests in parallel.

The sulfoxide index I(S=O), carbonyl index I(C=O) and butadiene index I(C=C) are defined as shown in Equations (2)–(4).

(2) $$I(S=O)=\frac{Sulfoxide\;based\;peak\;area(cenreredon\;1030\;cm^{-1})}{Peak\;area\;between\;2000-600\;cm^{-1}}$$

(3) $$I(C=O)=\frac{Carbonyl\;based\;peak\;area(cenreredon\;1700\;cm^{-1})}{Peak\;area\;between\;2000-600\;cm^{-1}}$$

(4) $$I(C=C)=\frac{Butadiene\;based\;peak\;area(cenreredon\;1600\;cm^{-1})}{Peak\;area\;between\;2000-600\;cm^{-1}}$$

### 4.2. Frequency Sweep Test

Frequency sweep tests were conducted on 70# and HCRA specimens with different degrees of ageing. According to the test specification, the rheological property indices, including the complex modulus (G*) and phase angle (δ), were measured at 46 °C~94 °C with a temperature interval of 6 °C. The temperature settings were used in the frequency sweep test because the high-temperature PG grades of 70# and HCRAs are PG64 and PG82, respectively, as shown in Table 2. This test was conducted using a DSR. A parallel loading plate with a diameter of 25 mm and a specimen gap of 1.0 mm was selected; the control strain was 0.1%, and the frequency sweep range was 0.1~100 rad/s.

### 4.3. Multiple Stress Creep Recovery (MSCR) Test

The MSCR test can be used to investigate the rheological properties of asphalt binder at high temperatures. Stress loading and unloading modes can be used; these modes are closely aligned with the loading of actual asphalt pavement. Researchers have indicated that there is a strong correlation between the MSCR test indicators of asphalt binder and the high-temperature performance of asphalt mixtures and pavement [44,45,46].

This experiment was conducted for 70#, HCRA35% and HCRA50% with different degrees of ageing at four stress levels, 0.1 kPa, 3.2 kPa, 6.4 kPa and 12.8 kPa, using DSR. Ten cycles were performed for each stress level, and each stress cycle comprised 1.0 s of loading followed by 9.0 s of unloading. Parallel loading plates (25 mm in diameter) with a gap of 1.0 mm were used. The temperature during the test was 64 °C. The test data were used to calculate the nonrecoverable creep compliance (J_nr_) and percent recovery (R%) to further characterize the high-temperature rheological properties of the asphalt binder specimens. The expressions for Jnr and R% are shown as Equations (5) and (6) [47]:

(5) $$J_{nr}=\frac{\varepsilon_{u}}{\sigma }$$

(6) $$R\%=\frac{\varepsilon_{p}-\varepsilon_{u}}{\varepsilon_{p}}\times 100\%$$

where $\varepsilon_{u}$ represents the nonrecovered strain, σ is the stress level and $\varepsilon_{p}$ represents the creep strain.

### 4.4. Linear Amplitude Sweep (LAS) Test

The LAS test can determine the fatigue properties of asphalt binder in a relatively short time. There are two primary parts of the LAS test. A strain load of 0.1 kPa is applied in the first section of the test, which involves a frequency sweep from 0.2 Hz to 30 Hz. The second section is an amplitude sweep test under controlled constant strain at 10 Hz, in which the applied strain load is linearly increased from 0.1% to 30%, with each strain lasting 10 s and 100 loading cycles per second for a total of 3100 cycles in the test [48]. The test was performed using a DSR with 8 mm loading plates with a spacing of 2 mm, and the temperature was set at 25 °C.

The resistance of the asphalt binders to fatigue damage can be evaluated using the LAS test. The fatigue damage of asphalt binder is characterized by the cumulative fatigue damage parameter D, as shown in Equation (7).

(7) $$D_{\left(t\right)}≅\sum_{i=1}^{N}{\left[\pi I_{D}\gamma_{0}^{2}\left(|G^{*}|\sin\delta_{i-1}\right)\right]}^{\frac{\alpha }{1+\alpha }}{\left(t_{i}-t_{i-1}\right)}^{\frac{1}{1+\alpha }}$$

where $I_{D}$ is the complex modulus at 1.0% strain; $\gamma_{0}$ is the strain; ${|G}^{*}|$ is the complex modulus; t is the test time; and the parameter α characterizes the rheological properties of the asphalt binder and it is calculated using Equations (8) and (9):

(8) $$\log G^{\prime }\left(\omega \right)=m\left(\log\omega \right)+b$$

(9) $$\alpha =1+\frac{1}{m}$$

where $G^{\prime }(\omega )$ is the storage modulus, $G^{\prime }\left(\omega \right)=|G^{*}|\cos\delta (\omega )$; ω is the angular frequency; and m is the fitting parameter.

The fitted relationship between $\left|G^{*}|\sin\delta (\omega \right)$ and D(t) is shown as Equation (10):

(10) $$|G^{*}|\sin\delta =C_{0}-C_{1}{\left(D\right)}^{C_{2}}$$

where $C_{0}=1$ and $C_{1}$ and $C_{2}$ are fitting parameters calculated from the power linearization of Equation (11):

(11) $$\log\left(C_{0}-|G^{*}|\sin\delta \right)=\log\left(C_{1}\right)+C_{2}\log\left(D\right)$$

## 5. Results and Discussion

### 5.1. Effect of Ageing on the Chemical Composition of the Asphalt Binders

Infrared absorption spectra can be employed to analyze the potential molecular structure of a given substance, identify functional groups within organic compounds and postulate possible chemical compositions. During the ageing process of asphalt binder, ketones and sulfoxides emerge as the primary oxidation products. In the FTIR spectroscopy, the carbonyl (C=O) absorption region predominantly comprises ketone-based functional groups, whereas sulfoxide groups (S=O) are primarily generated through the sulphides [49]. The primary alterations in the functional groups of HCRA take place in the spectral region associated with the stretching vibrations of sulfoxides (S=O) and butadiene groups (C=C) [50]. Furthermore, the carbonyl group exhibits peak absorbances in the range of 1800–1650 cm^−1^ [51]. the sulfoxide group displays peaks between 1050 and 950 cm^−1^, and the butadiene group shows peaks in the region of 1600–1450 cm^−1^. To assess the alterations in specific functional groups resulting from ageing, the ageing of HCRA is typically described by I(C=O), I(S=O), and I(C=C), which are defined as the measured peak areas of the respective bands at specific wavenumbers in the spectrogram [50].

**Figure 2:**
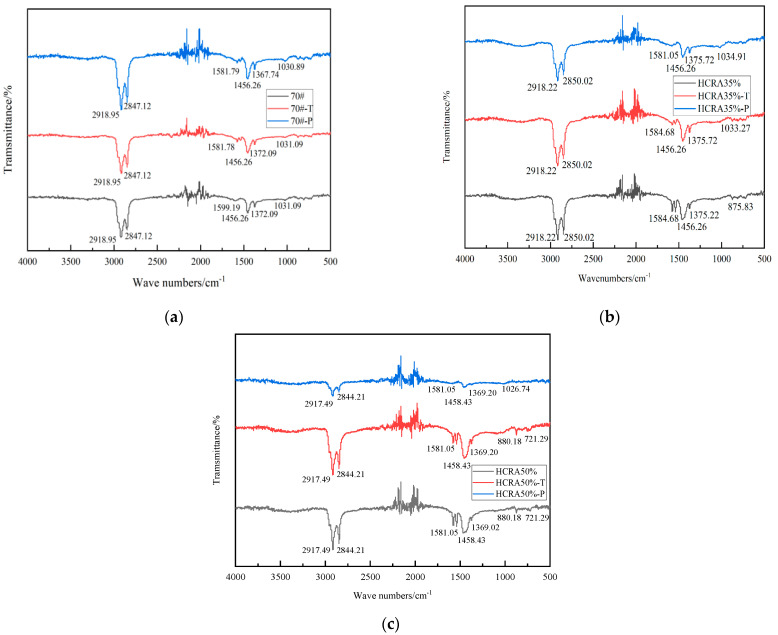
(**a**) Spectra results of 70# with different degrees of ageing. (**b**) Spectra results of HCRA35% with different degrees of ageing. (**c**) Spectra results of HCRA50% with different degrees of ageing.

As shown in Figure 2, both the 70# and HCRAs exhibit comparable spectral trends prior to and following ageing [52]. As shown in Figure 2b,c, after undergoing short-term and long-term ageing, in comparison with the 70# neat asphalt binder, the peak area of the butadiene group for HCRA35% and HCRA50% decreases due to a C=C bond fracture. The peak areas associated with carbonyl, sulfoxide and butadiene groups serve as three key indicators for characterizing the oxidation of asphalt binder, as these areas are closely linked to the extent of oxidation of the functional groups within the asphalt binder [48].

**Figure 3:**
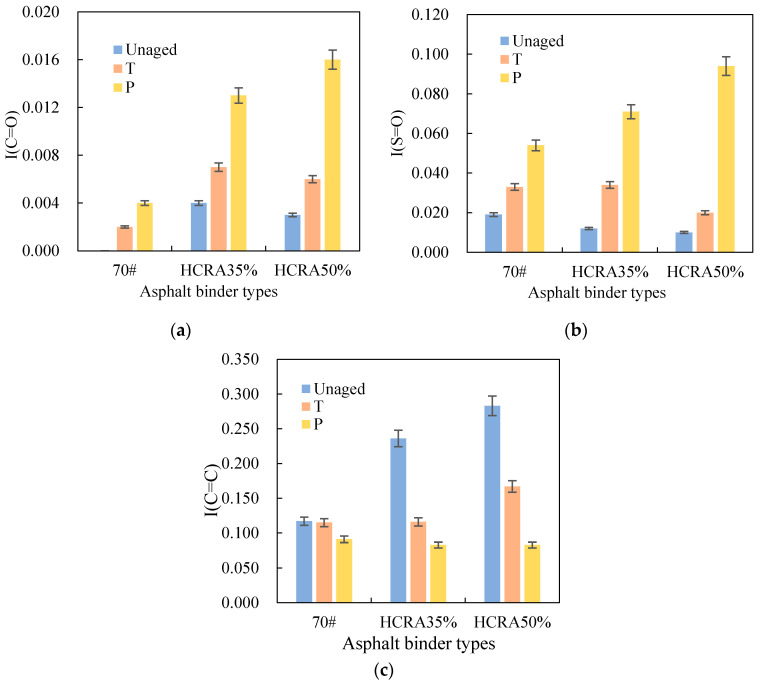
(**a**) Chemical indicators for 70# and HCRA (I(C=O)). (**b**) Chemical indicators for 70# and HCRA (I(S=O)). (**c**) Chemical indicators for 70# and HCRA(I(C=C)).

The results of changes in I(C=O), I(S=O) and I(C=C) for 70#, HCRA35% and HCRA50% at different degrees of ageing are shown in Figure 3. Figure 3a shows that the I(C=O) values of 70#, HCRA35% and HCRA50% increased with the degree of ageing. The I(C=O) of 70# increased to a lesser extent, but the I(C=O) of HCRA increased very significantly, especially after long-term ageing. The I(C=O) of HCRA35% increased faster than that of HCRA50% after short-term ageing. However, during long-term ageing, the I(C=O) of HCRA50% increased faster than that of HCRA35%. As shown in Figure 3b, the I(S=O) values of 70# and HCRA had similar trends to I(C=O) (in Figure 3a), which is consistent with the findings of previous studies [53]. The I(S=O) values of 70# and HCRA significantly increased during ageing, especially after long-term ageing. In addition, the incremental rates of I(S=O) values for HCRA35% and HCRA50% are greater than those of 70#.

As shown in Figure 3c, the I(C=C) values of 70# and HCRAs gradually decreased as the degree of ageing increased, but a small decrease was observed in 70#. The I(C=C) values of the HCRAs declined significantly. This might be attributed to the fact that ageing leads to the breaking of C=C bonds, resulting in a decrease in I(C=C). The I(C=C) values of HCRA35% and HCRA50% increased with increasing crumb rubber content at the same degree of ageing, possibly because the degradation of crumb rubber during ageing broke the rubber molecular chain, which caused a drop in the absorbance of the characteristic peaks [49]. Figure 3c shows that the rate of change in these three indicators, including I(C=O), I(S=O) and I(C=C), with ageing was greater for HCRAs than for 70#, generally. The I(C=C) is more suitable than the other two indicators for characterizing the ageing behaviour of HCRA.

### 5.2. Effect of Ageing on the Rheological Properties of Asphalt Binder

#### 5.2.1. Frequency Sweep Test Results

The frequency sweep test yielded the indices of G* and δ for 70#, HCRA35% and HCRA50%. G* and δ are two important rheological property parameters describing the viscoelastic characteristics of asphalt binder. G* describes the ability of asphalt binder to resist deformation under repeated loading in specific circumstances. The higher G* is, the better the resistance of the asphalt binder to deformation at high temperatures [54]. δ can be used to evaluate the proportion of viscoelastic components in asphalt binder. In asphalt binders, a larger δ indicates that viscous characteristics dominate; in contrast, a lower δ suggests that the elasticity characteristics dominate. In the latter case, the occurrence of permanent deformation under a load can be reduced.

**Figure 4:**
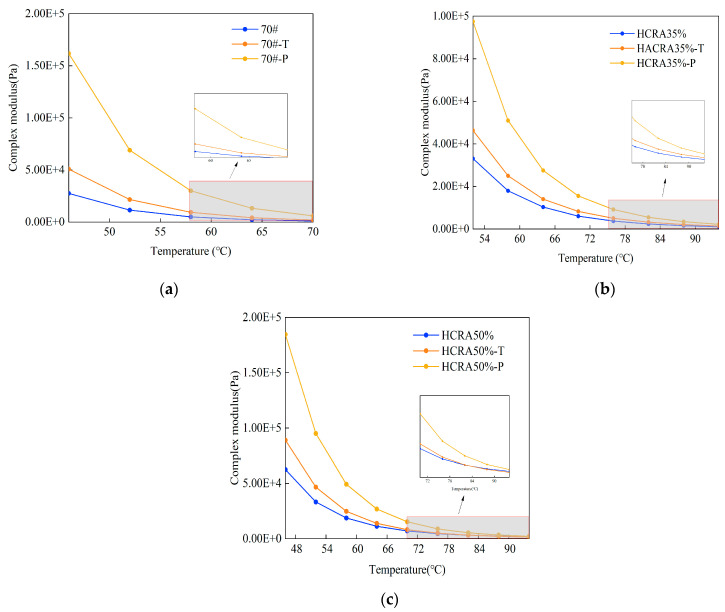
(**a**) Complex modulus of 70#. (**b**) Complex modulus of HCRA35%. (**c**) Complex modulus of HCRA50%.

Figure 4a–c present the G* values of 70#, HCRA35% and HCRA50% at different ageing degrees. It can be seen that, at the same temperature, the G* value for 70# increases with ageing, and HCRAs show the same trend. HCRA gradually hardened with ageing, as did 70#. The G* values of both 70# and HCRAs gradually decreased with increasing temperature at the same degree of ageing. The temperature sensitivity of 70# and HCRAs decreased when the temperature increased. In addition, their temperature sensitivity increased during ageing when the temperature was below 64 °C, especially for the HCRA35% and HCRA50%. The values of G* and their changing trend were similar for HCRA35% and HCRA50%. In addition, the G* of HCRA with the addition of crumb rubber was always larger than that of 70#, indicating that the addition of crumb rubber can improve the deformation resistance of asphalt binder. This was particularly evident in the unaged asphalt binder specimens.

**Figure 5:**
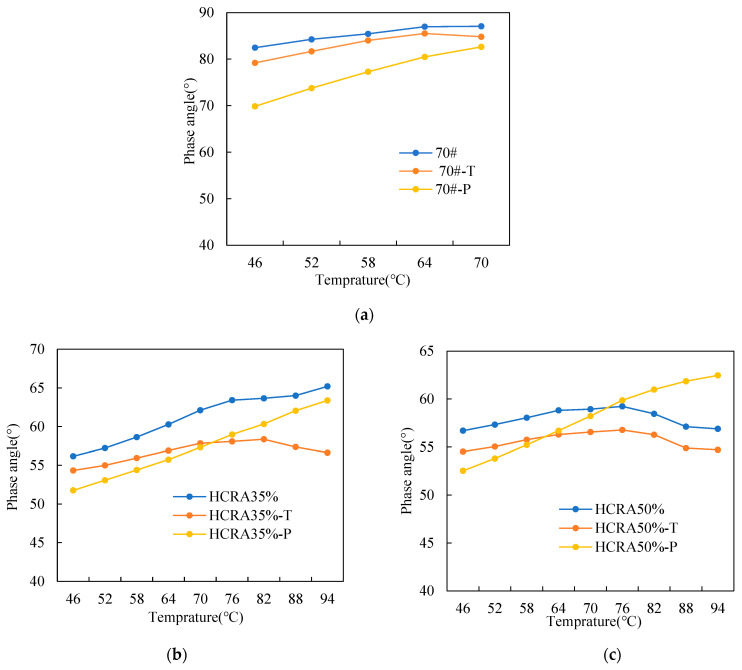
(**a**) Phase angle of 70#. (**b**) Phase angle of HCRA35%. (**c**) Phase angle of HCRA50%.

As shown in Figure 5, in general, the value of δ increased with increasing temperature. In addition, the δ value for 70# with different degrees of ageing was much greater than that of HCRA. Clearly, 70# showed more highly viscous characteristics. This indicates that the viscous characteristic of asphalt binder decreased by blending the asphalt binder with crumb rubber and its deformation recovery ability improved.

In Figure 5a, for 70#, the δ value decreased after ageing, especially for the long-term ageing. Therefore, ageing converted part of the asphalt binder from viscous to elastic. In addition, the temperature sensitivity of 70# increased after long-term ageing. The difference in δ values between aged and unaged 70# was smaller with increasing of temperature.

As shown in Figure 5b,c, the δ values for HCRA35% and HCRA50% showed different trends. The δ values for HCRA35% and HCRA50% decreased when they were during short-term ageing. However, after long-term ageing, the δ values for HCRA35% and HCRA50% presented an increasing trend compared to those of short-term ageing in the high-temperature region. This may be because the degradation of crumb rubber during long-term ageing promoted the softening of the asphalt binder, and subsequently, when the temperature increased, the viscous component of HCRA became more significant. Furthermore, the increase in δ values for long-term-aged HCRA50% was more pronounced compared to that of long-term-aged HCRA35%. This result indicates that under long-term ageing, a higher content of crumb rubber softens the asphalt binder more, thereby compensating for the hardening effect of the ageing process [55].

#### 5.2.2. Results of MSCR Test

The MSCR test was used to characterize the linear and nonlinear high-temperature viscoelasticity of asphalt binders and to determine the rutting potentials of the asphalt binder by comparing the magnitudes of Jnr and R%.

**Figure 6:**
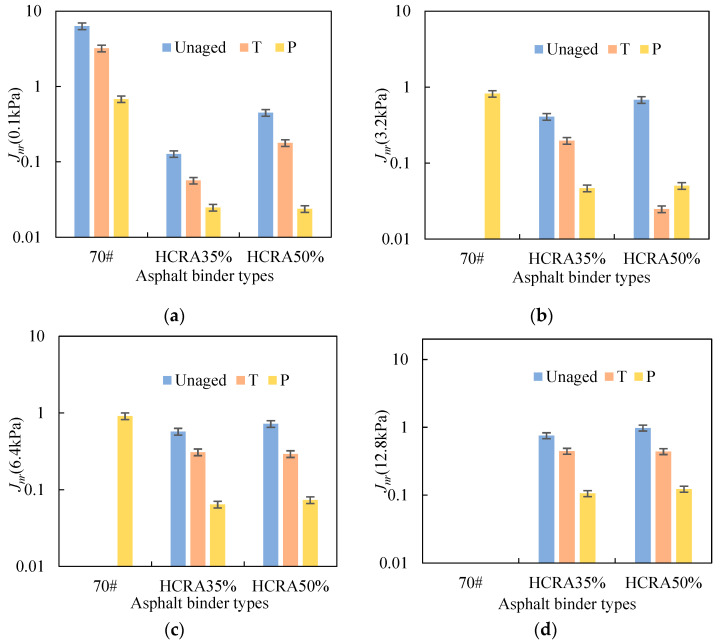
Jnr values of 70# and HCRAs under different degrees of ageing: (**a**) 0.1 kPa; (**b**) 3.2 kPa; (**c**) 6.4 kPa; (**d**) 12.8 kPa.

Figure 6a–d show the Jnr values of 70# and HCRAs under different degrees of ageing calculated according to Equation (5). Due to the poor high-temperature viscoelastic properties of 70#, the Jnr values of unaged and short-term-aged 70# could only be measured from the effective results at 0.1 kPa. The Jnr values of long-term-aged 70# were measured at 0.1 kPa, 3.2 kPa and 6.4 kPa. In general, 70# had a much greater Jnr than HCRA35% and HCRA50% under the same stress loading. It is evident that 70# has a poor rutting resistance and is prone to permanent deformation at high temperature. It is clear that the inclusion of crumb rubber in HCRA improves the rutting resistance performance of the asphalt binder. Furthermore, as the degree of ageing increased, the Jnr values of 70#, HCRA35% and HCRA50% decreased, respectively, under the same loading stress. Evidently, the ageing response of HCRA was similar to that of 70#, with the hardening of the asphalt binder and an improvement in the rutting resistance being more pronounced after ageing. For the Jnr results of HCRA35% and HCRA50%, the rutting resistance of HCRA35% was slightly better than that of HCRA50%, particularly under low-stress conditions. This suggests that a high content of crumb rubber does not necessarily improve the ageing behaviour of the HCRA.

**Figure 7:**
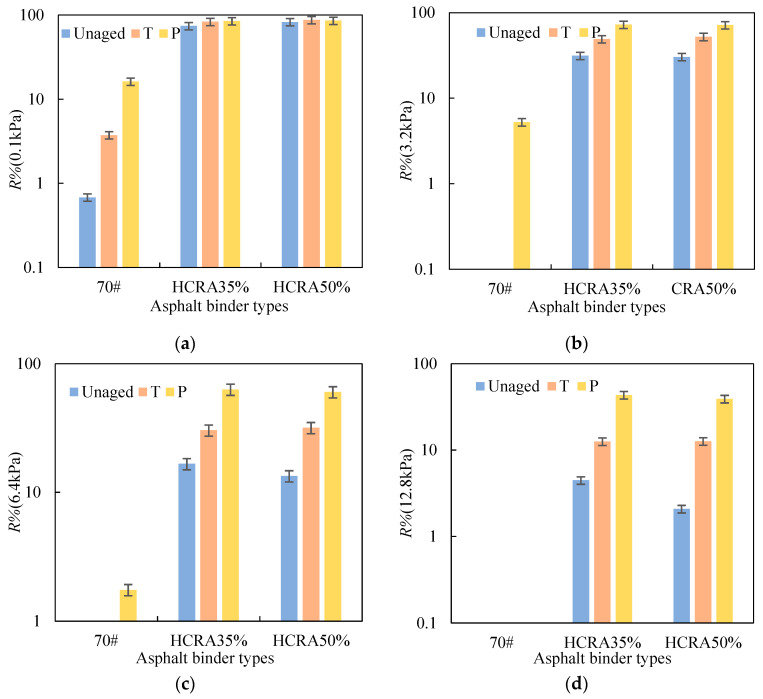
R% values of 70# and HCRA under different degrees of ageing: (**a**) 0.1 kPa; (**b**) 3.2 kPa; (**c**) 6.4 kPa; (**d**) 12.8 kPa.

The *R%* values of 70# and HCRA with different ageing degrees were calculated according to Equation (6), and the results are shown in Figure 7a–d. Similarly to the results of *J_nr_*, the *R*% values for unaged and short-term aged 70# were calculated at 0.1 kPa, while the *R*% values for long-term aged 70# were determined at 0.1 kPa, 3.2 kPa and 6.4 kPa. As shown in Figure 7, as the degree of ageing increased, the *R*% values for 70# and HCRA gradually increased, indicating that the asphalt binders exhibited stronger elastic characteristics. This is attributed to the hardening of the asphalt binder after ageing. Furthermore, HCRA exhibited a better elastic recovery ability than 70#. The blending of crumb rubber proved effective in enhancing the elastic properties of the asphalt binder. When considering the results of *R*% for HCRA35% and HCRA50%, they exhibited great elastic recovery abilities at 0.1 kPa, despite undergoing short-term and long-term ageing. As the applied stress increased, a significant difference in *R*% emerged among the unaged, short-term aged and long-term aged HCRAs. This observation suggests that relatively high-stress conditions should be employed when investigating the ageing properties of the HCRA.

#### 5.2.3. Results of the LAS Test

**Figure 8:**
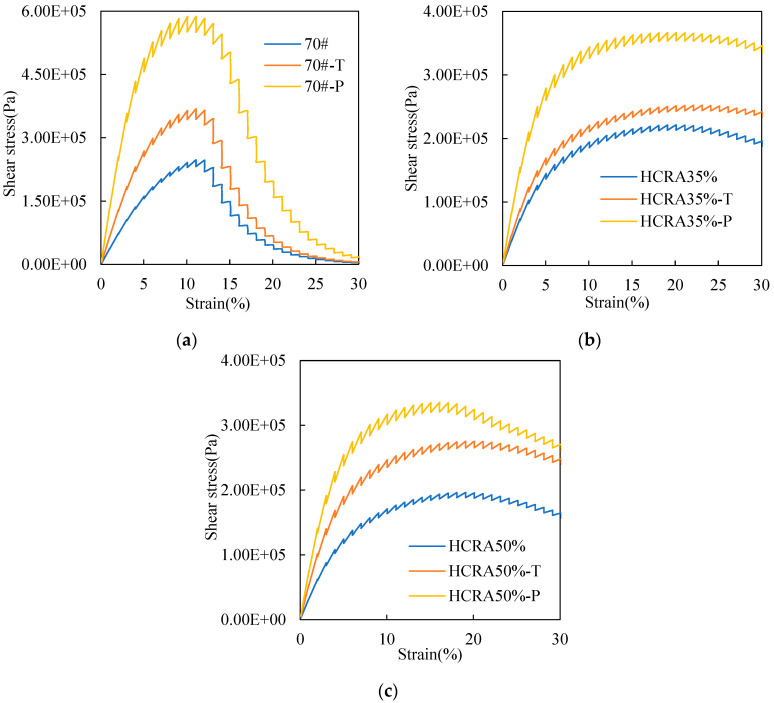
(**a**) Stress–strain of 70# at different degrees of ageing. (**b**) Stress–strain of HCRA35% at different degrees of ageing. (**c**) Stress–strain of HCRA50% at different degrees of ageing.

Recently, a combination of LAS tests and viscoelastic continuum damage (VECD) theory has been successfully applied in the fatigue evaluation of asphalt binders [56]. In this study, the stress–strain curve of 70#, HCRA35% and HCRA50% with different ageing degrees can be obtained using the LAS test. The results are shown in Figure 8a–c. The stress–strain curves for 70#, HCRA35% and HCRA50% exhibited a similar trend, with shear stress reaching a peak and subsequently decreasing, featuring an inflexion point. This is due to the increase in shear strain as a result of damage to the asphalt binder.

Ageing leads to a peak in shear stress in asphalt. For both 70# and HCRA, the overall trend remains consistent: the peak stresses observed after long-term ageing significantly exceed those seen in short-term ageing and unaged conditions. Consequently, the stress–strain tolerance of the asphalt binder diminishes under heavy loads. On the whole, at the same ageing level, 70# and HCRA display comparable trends, characterized by an initial increase followed by a decrease. As the shear strain intensifies, the shear stress of 70# diminishes to 0. Conversely, HCRA maintains a notably high stress level even under shear strain as high as 30%, demonstrating its robust structural integrity. This observation aligns with previous research findings [55]. Figure 8 also reveals distinct differences in the curve shapes of 70# and HCRA. A larger area in the peak region indicates that the asphalt binder exhibits a lower sensitivity to strain under shear loading at a given rate, thereby implying a higher fatigue performance. The peak area of the stress–strain curve for HCRA is greater than that of 70#, suggesting that HCRA has a lower sensitivity to shear strain compared to 70#. Consequently, HCRA’s fatigue performance surpasses that of 70#. Additionally, the peak areas for HCRA35% and HCRA50% are quite similar, with HCRA35% exhibiting a slightly better fatigue performance than HCRA50%.

**Figure 9:**
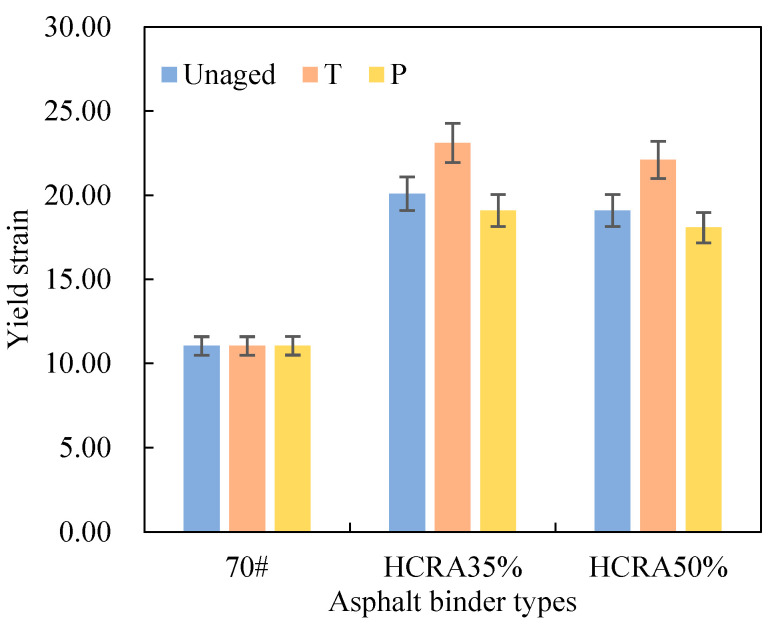
Yield strain of asphalt binders.

When the asphalt attains a certain stress level, a stress peak emerges, which is referred to as the fatigue failure point. The corresponding fatigue strain is known as the yield stress [48], a value that reflects the fatigue performance. As shown in Figure 9, the yield strains of 70# and HCRA under various ageing conditions have been calculated. The yield strain of 70# remains relatively constant with increasing age, indicating that 70# is not highly sensitive to ageing.

Conversely, as the degree of ageing increases, the yield strain of HCRA initially rises and then declines. Therefore, an appropriate level of short-term ageing can enhance HCRA’s fatigue performance, as the degradation of rubber crumb in the short term can counteract the hardening of the asphalt binder. However, after long-term ageing, HCRA’s fatigue performance notably decreases to the level of unaged HCRA. Furthermore, under the same ageing conditions, HCRA’s yield strain is greater than that of 70#, indicating that the fatigue performance of asphalt binder can be significantly improved by the addition of rubber crumb. A comparison of the yield strains of HCRA35% and HCRA50% reveals that HCRA35% exhibits a better fatigue performance than HCRA50%.

**Figure 10:**
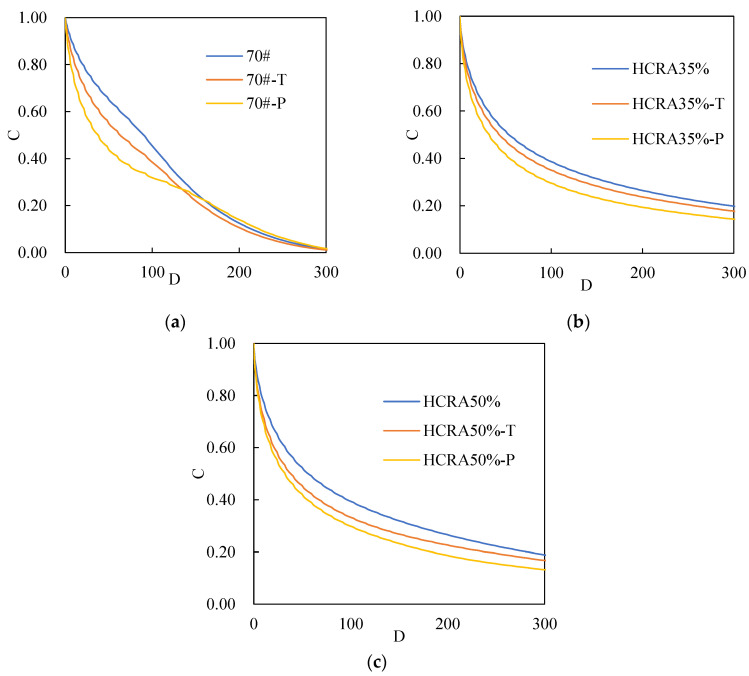
(**a**) C–D curves of 70# and HCRA with different degrees of ageing. (**b**) C–D curves of HCRA35% with different degrees of ageing. (**c**) C–D curves of HCRA50% with different degrees of ageing.

Figure 10 shows the fatigue failure curve of asphalt binder samples obtained using the VECD model, where the horizontal axis (D) represents the cumulative damage parameter of the asphalt binder, and the vertical axis (C) denotes the integrity parameter [57]. When C equals 0, the asphalt binder is in a state of complete failure. Conversely, when C equals 1, the asphalt binder is in a pristine, undamaged state [48].

As shown in Figure 10a–c, both 70# and HCRA exhibit similar downward trends in their integrity parameter C. When the damage parameter D reaches 300, the integrity parameter C of 70# drops to 0, indicating the complete failure of 70#. However, the integrity parameter C of HCRA remains around 0.2, suggesting that the addition of rubber crumb enhances the asphalt binder’s resistance to damage. It is evident that long-term ageing has a more significant impact on the damage to asphalt. Nevertheless, as the damage parameter D increases to approximately 150, the integrity parameter C of long-term aged 70# shows a tendency to increase. A comparison of the integrity parameter C values for HCRA35% and HCRA50% reveals that HCRA35% exhibits a stronger resistance to damage.

**Figure 11:**
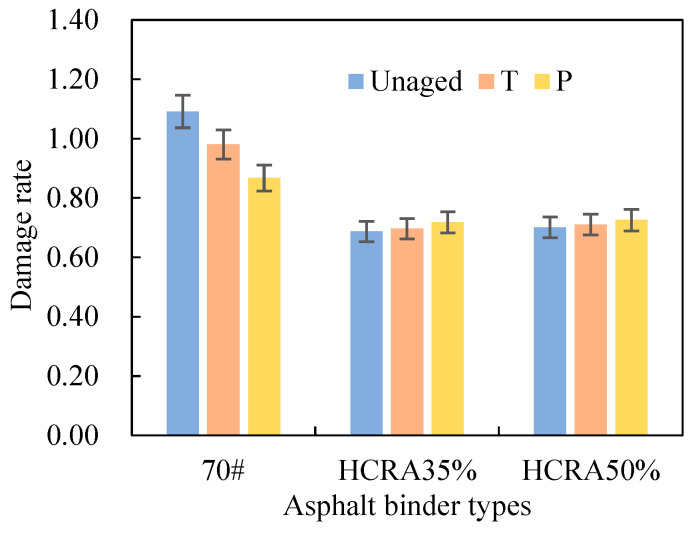
Damage rates of asphalt binders.

The rate of decrease in the virtual modulus of the asphalt binder, also known as the damage rate, can be determined based on the slope of the C–D curve [48]. As illustrated in Figure 11, the damage rate of 70# decreases with increasing ageing. However, the damage rate of HCRA gradually increases, with HCRA35% and HCRA50% exhibiting similar trends. This may be attributed to ageing causing an increase in the stiffness of 70#, temporarily enhancing its resistance to damage. In terms of the damage rate, HCRA35% exhibits a slightly higher resistance to damage than HCRA50%. This finding is consistent with the conclusions drawn from the stress–strain analysis, although the differences are not significant.

### 5.3. Ageing Sensitivity of HCRA

The chemical composition and rheological properties determined from the above experiments showed that both short- and long-term ageing affect the performance of asphalt binders. To more intuitively study the impact of ageing on asphalt binders, the ageing index (AI) was used to characterize the binders. A higher AI indicates greater ageing sensitivity. The expression for AI is as follows:

(12) $$AI=\frac{Aged-Unaged}{\left|Unaged\right|}$$

where AI is the ageing index, Aged is the value of a characteristic parameter when the asphalt binder is aged, and Unaged is the value of a characteristic parameter when the asphalt binder is unaged.

As shown in Figure 12, Figure 13, Figure 14, Figure 15, Figure 16, Figure 17, Figure 18 and Figure 19, the AIs of G*, δ, Jnr, R%, peak stress, C-slope and yield strain corresponding to different ageing degrees were calculated using Equation (12).

#### 5.3.1. Viscoelastic Properties

**Figure 12:**
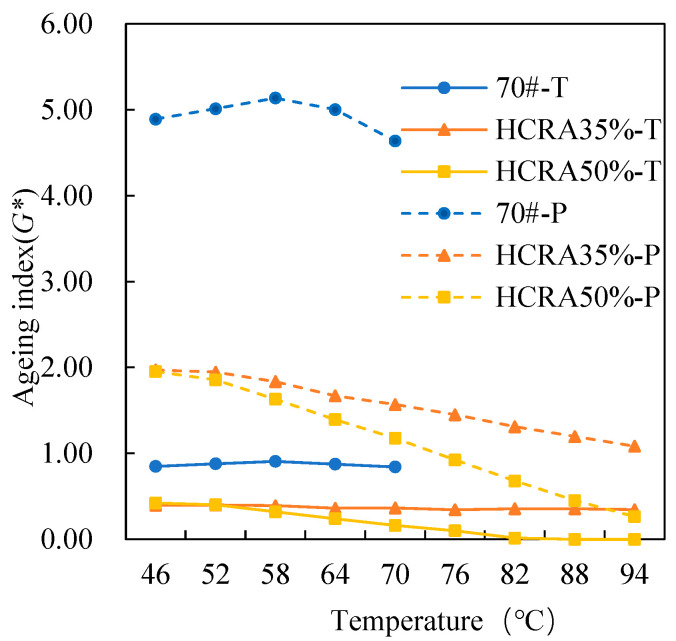
The G* AI of the rheological properties of 70# and HCRA.

Figure 12 compares the AI-G* values of 70# and HCRA during short-term ageing. It is evident that 70# > HCRA35% > HCRA50%, indicating that 70# is more sensitive to ageing. Consequently, the addition of rubber crumb significantly improves the ageing resistance of the asphalt. The trend for long-term ageing is consistent with that for short-term ageing, but overall, both 70# and HCRA exhibit higher sensitivity to long-term ageing compared to short-term ageing. In the low-temperature region, HCRA35% and HCRA50% display a similar ageing sensitivity. However, as the temperature rises, HCRA50% exhibits a better ageing resistance.

**Figure 13:**
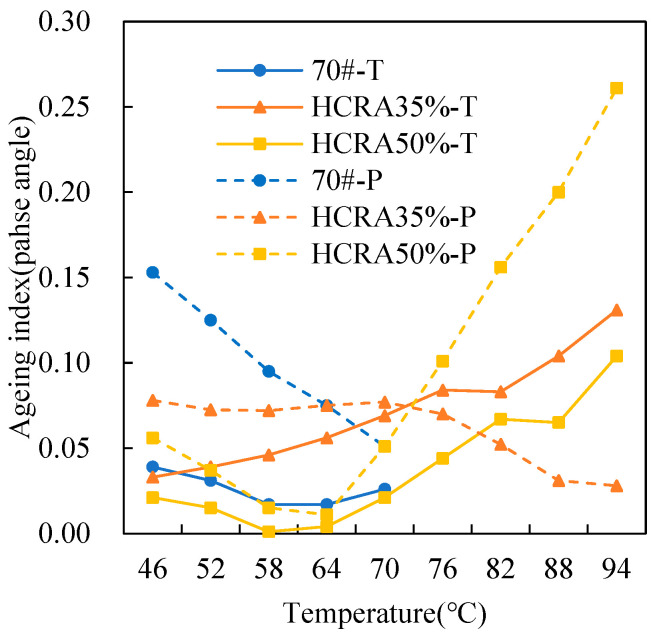
The *δ* AI of the rheological properties of 70# and HCRA.

As shown in Figure 13, similar to AI-G*, AI-δ is also highly sensitive to temperature. In the low-temperature region, under short-term ageing conditions, the AI-δ values of 70# and HCRA follow the order HCRA35% > 70# > HCRA50%. However, after long-term ageing, the AI-δ value of 70# exceeds that of HCRA35% and HCRA50%. There is a noticeable decreasing trend in the AI-δ of 70#, possibly due to the dual effects of temperature and ageing, which enhance its resistance to deformation. Nevertheless, HCRA demonstrates clear advantages. In the high-temperature region, the AI-δ of HCRA35% gradually decreases, whereas the AI-δ of HCRA50% shows a significant increasing trend. This result may be attributed to the higher content of rubber crumb in HCRA50%. At high temperatures, the degradation of rubber crumb increases the viscoelasticity of HCRA, leading to an increase in AI-δ and a decrease in ageing resistance for HCRA50%.

#### 5.3.2. High-Temperature Performance

**Figure 14:**
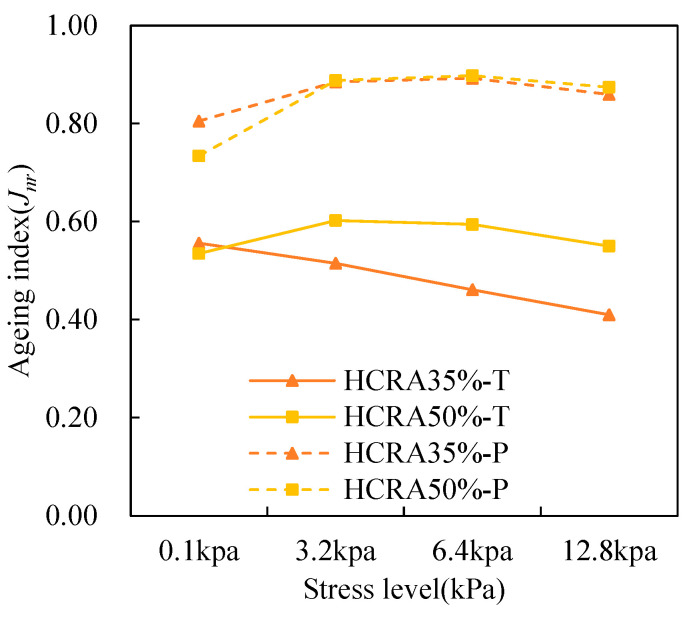
The *Jnr* AI values of the rheological properties of HCRA.

Jnr and R% are employed to characterize the high-temperature properties of asphalt binders [55]. As illustrated in Figure 14, after long-term ageing, the difference in AI-Jnr between HCRA35% and HCRA50% diminishes, whereas this difference is reversed during short-term ageing. This finding indicates that the resistance to deformation of HCRA is highly sensitive to ageing, and long-term ageing exacerbates the permanent deformation of HCRA under repeated loading. A comparison of the AI-Jnr values for HCRA35% and HCRA50% reveals that HCRA35% exhibits stronger resistance to ageing. This result contrasts with the conclusions drawn from the AI-G* results, potentially due to G* being measured under linear loading conditions, whereas Jnr is measured under nonlinear loading conditions.

**Figure 15:**
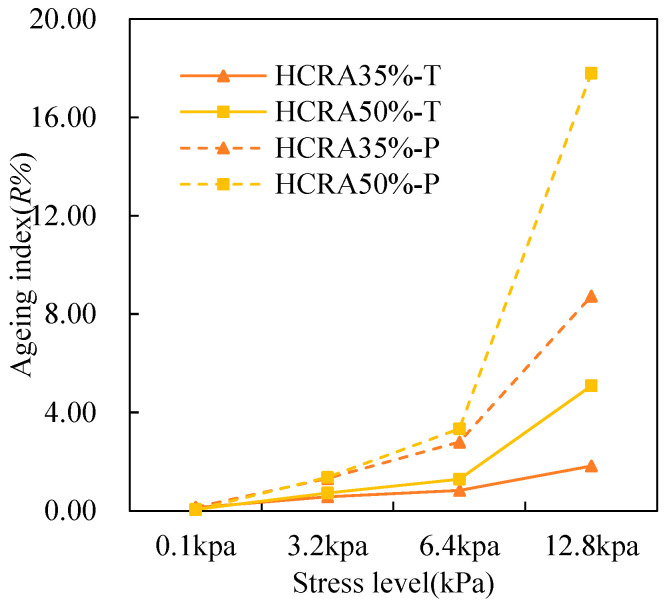
The *R*% AI values of rheological properties of HCRA.

R% is used to characterize the recovery properties of asphalt binders, and the results indicate that the stress level has a significant impact on these properties. As shown in Figure 15, for HCRA, the sensitivity of AI-R% to stress is greater than that of AI-Jnr. At the same stress level, ageing enhances its elastic properties. However, for unaged asphalt binders, as shown in Figure 15, higher stress levels lead to smaller R% values, resulting in larger AI-R% values. From the perspective of recovery properties, HCRA35% exhibits better resistance to ageing.

#### 5.3.3. Fatigue Performance

**Figure 16:**
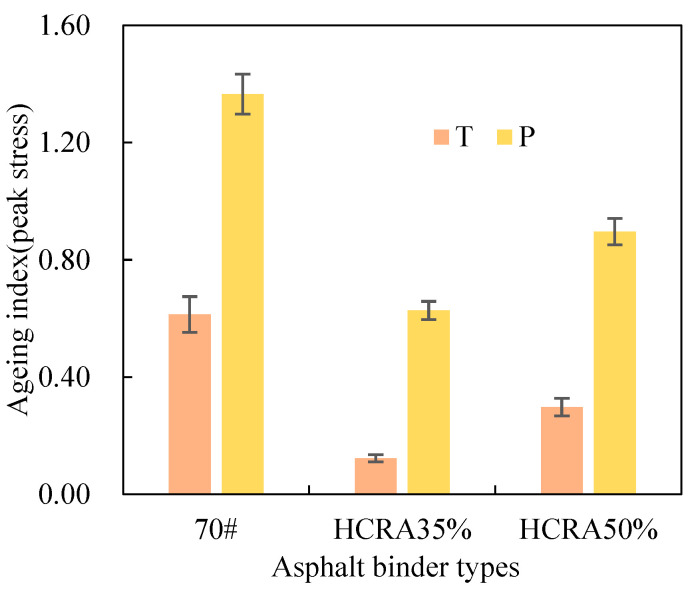
Peak stress AI of rheological properties of 70# and HCRA.

As shown in Figure 16, during the short-term ageing process, the order of peak stress AIs for 70# and the HCRA samples is 70# > HCRA50% > HCRA35%. The peak stress AI of 70# is significantly greater than that of HCRA. A similar trend is observed during the long-term ageing process. However, the peak stress AI for long-term ageing is substantially higher than that for short-term ageing. This indicates that the load-bearing capacity is more sensitive to long-term ageing. An increase in the degree of ageing reduces the load-bearing capacity of the asphalt binder. It is evident that the load-bearing capacity of HCRA is greater than that of 70#. A comparison of the peak stress AI between HCRA35% and HCRA50% reveals that HCRA35% exhibits better resistance to ageing.

**Figure 17:**
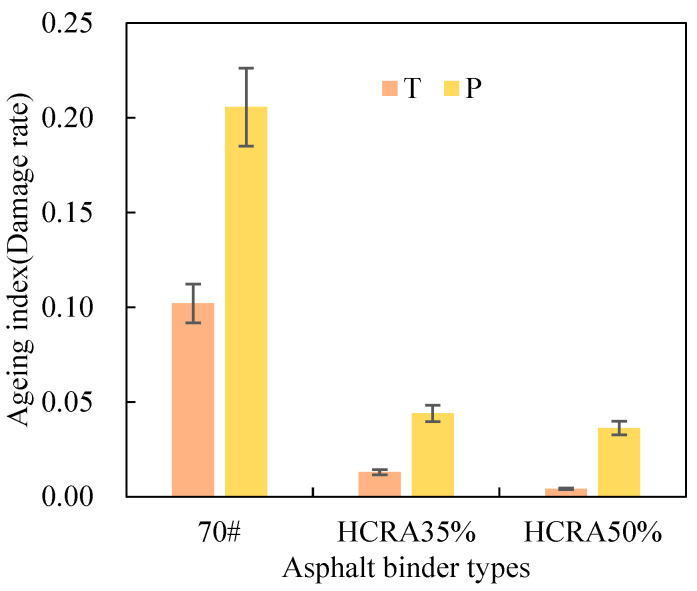
Damage rate AIs of rheological properties of 70# and HCRA.

As illustrated in Figure 17, the damage rate AI of 70# surpasses that of HCRA by a considerable margin, irrespective of whether the ageing process is short-term or long-term. The sensitivity of HCRA’s damage rate to ageing is markedly inferior to that of 70#. Furthermore, the damage rate of asphalt binders exhibits a heightened sensitivity to long-term ageing compared to short-term ageing. Upon comparing the damage rate AI values of HCRA35% and HCRA50%, it is evident that, from the vantage point of the damage rate, HCRA50% demonstrates a superior resistance to ageing.

**Figure 18:**
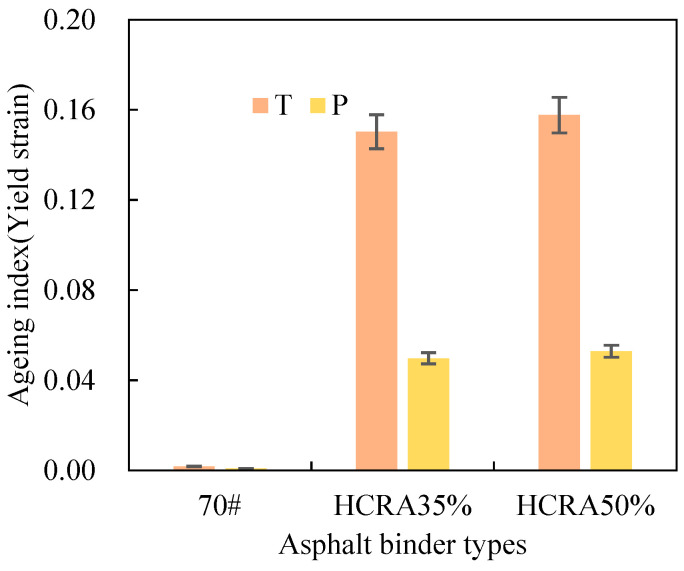
AIs of rheological properties of 70# and HCRA (peak stress, damage rate, and yield strain).

From Figure 18, it can be inferred that the yield strain AI of 70# under varying degrees of ageing remains relatively constant, with no discernible trend observed. Conversely, for HCRA, there is a pronounced trend in the yield strain AI. Both HCRA35% and HCRA50% exhibit a similar gradual decrease in their values during both short-term and long-term ageing processes. However, from the perspective of fatigue performance, long-term-aged HCRA demonstrates superior resistance to ageing. A comparison of the yield strain AI values for HCRA35% and HCRA50% indicates that HCRA35% performs slightly better. Nevertheless, it is not feasible to ascertain the sensitivity of the fatigue performance of 70# and HCRA to ageing based on this isolated dataset alone.

### 5.4. Correlation of Chemical Composition and Rheological Properties

**Figure 19:**
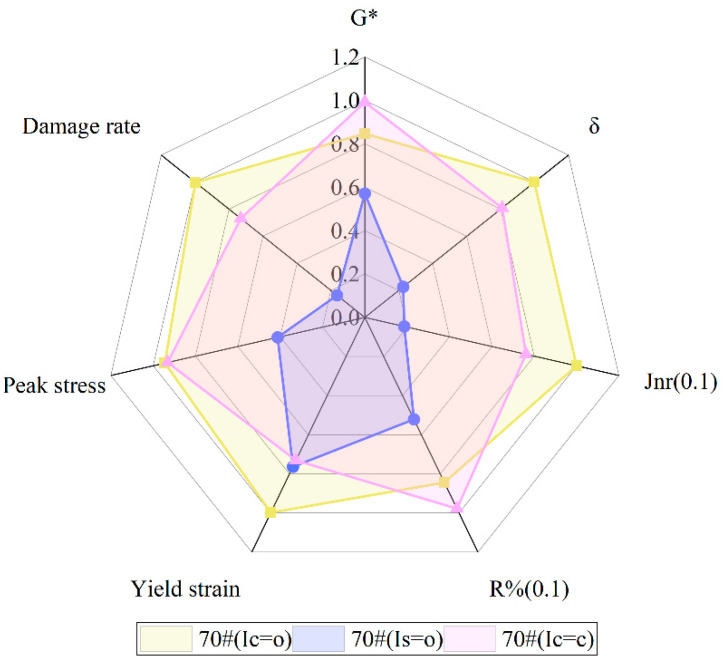
Correlation between chemical composition and rheological properties of asphalt binder.

To delve deeper into the correlation between the chemical indices and rheological properties of 70# and HCRA, a correlation analysis was conducted, as shown in Figure 19. The carbonyl (C=O) group at 1700 cm^−1^ and the sulfoxide (S=O) group at 1030 cm^−1^, both being oxidative groups, are the most pertinent indicators of asphalt ageing. Following long-term ageing, there is a notable increase in the quantity of carbonyl and sulfoxide groups within the asphalt. The area index serves as a reflection of the ageing extent of the internal components and structure of the asphalt binder. Desulfurized HCRA causes a significant change in the absorbance of butadiene (C=C) molecules located at 1600 cm^−1^. By lever ageing these three chemical indices, a comprehensive characterization of the internal component and structural changes in the asphalt binder can be achieved. Hence, in this study, chemical analytical methods were employed to calculate the area indices of the carbonyl (C=O), sulfoxide (S=O) and butadiene (C=C) groups. The rheological indices were obtained through a series of Dynamic Shear Rheometer (DSR) tests, encompassing G*, δ, Jnr, R%, peak stress, yield stress and damage rate. A correlation was established between these three chemical indices and the rheological performance indicators of 70# and HCRA.

**Figure 20:**
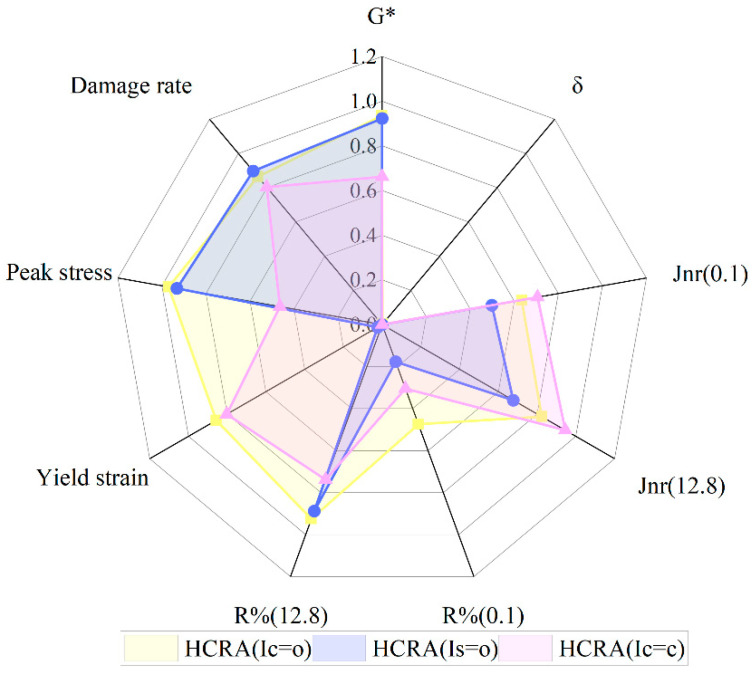
Correlation between chemical composition and rheological properties of asphalt binder.

As shown in Figure 20, for HCRA, there exists a significant correlation between I(C=O) and I(S=O) and G*, the damage rate, and Jnr and R% under high-stress conditions. The correlation between the three chemical characteristics and the rheological properties is comparable for both HCRA and 70#. No single chemical index emerges as the most strongly correlated. Nevertheless, an intriguing phenomenon is observed in Figure 20: all three chemical indices exhibit no correlation with δ, while their correlation with Jnr and R% is weak at low stress levels but strong at high stress levels. This finding may be attributed to the formation of a network structure within the polymer in HCRA, which affects its chemical composition and rheological properties. However, further verification is required to substantiate this conclusion.

## 6. Conclusions

In this study, the effects of short-term ageing and long-term ageing on the chemical composition changes and rheological properties of 70# and HCRA were investigated mainly by FTIR and DSR tests, and the relationship between them was analyzed by correlation analysis. Based on the experimental results, the following conclusions can be drawn:

1. The FTIR analysis revealed that the infrared spectrograms of all asphalt samples remained largely unchanged. However, a progressive increase in I(C=O) and I(S=O) was observed for both 70# and HCRA as ageing progressed, with this trend becoming more evident after long-term ageing. Conversely, I(C=C) exhibited a decreasing trend. In the case of HCRA, as the crumb rubber content increased, the oxidation of carbonyl and sulfoxide groups was found to decelerate, while the increase in I(C=C) was less pronounced. These findings suggest that while ageing affects the chemical indices of both 70# and HCRA, the presence of crumb rubber in HCRA may mitigate the oxidation process.
2. DSR tests revealed that ageing has a hardening effect on the asphalt binder, thereby altering the rheological performance characteristics of both 70# and HCRA. Specifically, ageing results in an elevation of G* and a reduction in δ for 70#. In contrast, for HCRA, δ decreases in the low-temperature region but increases in the high-temperature region due to ageing. Furthermore, as the ageing process intensifies, Jnr progressively diminishes, while R% gradually rises in the asphalt binder. Complementary to these findings, the LAS test, which assessed peak stress, the C-D curve, and other indicators, demonstrated that the incorporation of rubber crumb notably enhances the fatigue performance of the asphalt binder.
3. The ageing index (AI) was calculated using a specific expression to analyze the rheological property index. Based on this AI, the sensitivity of the rheological properties of 70# and HCRA to ageing was assessed. Additionally, a regression model was developed to establish a relationship between chemical composition and rheological property indicators. This model successfully predicts the rheological properties of both 70# and HCRA using three chemical indicators.
4. In conclusion, when comparing asphalt binder with crumb rubber to 70#, it is evident that the former exhibits superior viscoelastic properties, high-temperature performance, fatigue resistance and anti-ageing characteristics.

## Figures and Tables

**Table 1:** Composition of the crumb rubber.

Items	Ash (%)	Acetone Extract (%)	Rubber Hydrocarbon (%)	Carbon Black (%)
Measured results	8.0	8.0	56.0	29.0

**Table 2:** Properties of 70# and HCRAs.

Indicators	70#	HCRA35%	HCRA50%
Penetration (0.1 mm, 100 g, 5 s, 25 °C)	71	50	49
Ductility (cm, 5 cm/min, 5 °C)	0.3	72	75
Softening point (°C)	46.4	66.9	65.9
High-temperature performance grading (PG)	PG64	PG82	PG82

## Data Availability

Data are contained within the article.
